# Synthesis of Oxidant Functionalised Cationic Polymer Hydrogel for Enhanced Removal of Arsenic (III)

**DOI:** 10.3390/gels7040197

**Published:** 2021-11-04

**Authors:** Yu Song, Takehiko Gotoh, Satoshi Nakai

**Affiliations:** Laboratory of Polymer Engineering, Department of Chemical Engineering, Faculty of Engineering, Hiroshima University, Hiroshima 7398527, Japan; d215240@hiroshima-u.ac.jp (Y.S.); sn4247621@hiroshima-u.ac.jp (S.N.)

**Keywords:** hydrogel, oxidant, arsenic removal, Langmuir model, kinetics, co-existing ions

## Abstract

A cationic polymer gel (*N*-[3-(dimethylamino)propyl]acrylamide, methyl chloride quaternary)(DMAPAA-Q gel)-supported oxidising agent (KMnO_4_ or K_2_Cr_2_O_7_) was proposed to remove As from water. The gel could adsorb arsenite, As(III), and arsenate, As(V), through the ion exchange method, where the oxidising agent oxidised As(III) to As(V). theoretically speaking, the amount of oxidant in the gels can reach 73.7 Mol%. The maximal adsorption capacity of the D-Mn gel (DMAPAA-Q gel carrying MnO_4_^−^) and D-Cr gel (DMAPAA-Q gel carrying Cr_2_O_7_^2−^) for As(III) could reach 200 mg g^−1^ and 263 mg g^−1^, respectively; moreover, the As(III) removal rate of the gels could still be maintained above 85% in a neutral or weak acid aquatic solution. Studies on the kinetic and adsorption isotherms indicated that the As adsorption by the D-Mn and D-Cr gels was dominated by chemisorption. The thermodynamic parameters of adsorption confirmed that the adsorption was an endothermic process. The removal of As is influenced by the co-existing high-valence anions. Based on these results, the gels were found to be efficient for the As(III) adsorption and could be employed for the As(III) removal from the industrial wastewater.

## 1. Introduction

In the recent years, the reports of As contamination exceeding the environmental and wastewater standards in industrial wastewater have been increasing worldwide [1,2,3,4,5,6]. According to the latest fact sheets from WHO [7], tens of millions of people have consumed high-concentration As-contaminated groundwater [8,9]; health problems such as skin cancer caused by the As contaminants have become a major social problem [2,10,11]. Therefore, the development of advanced materials or technologies for the efficient treatment of As wastewater continues to be a global research priority. Various removal methods have been developed and applied to treat the As-contaminated wastewater, such as co-precipitation for the removal of arsenous acid using Fe(III) and Mn oxide, adsorption methods using various adsorbents and minerals, and ion exchange methods [4,5,12,13,14]. Among them, adsorption is widely used because of its technological simplicity, high efficiency, and low secondary pollution risk [15]. However, some conventional adsorbents have many drawbacks, such as high cost, few adsorbent sites, and small absorption capacity, which limits their application in the water treatment. Moreover, some conventional adsorbents have a low removal efficiency towards the highly toxic As(III), which further limits their application in water treatment [16].

Among the many adsorbents developed and used for As(III) removal, the application of polymer sorbents has been the most popular approach for As(III) removal from industrial wastewater, owing to their environmental safety, superior chemical modifiability, many adsorbent sites, and outstanding renewability [17,18,19]. There are two main types of polymer adsorbents, one of which adsorbs via electrostatic interactions through its cationic functional groups, while the other adsorbs by a functional group that has a high affinity for As. Among the cationic polymers [20], the polymers with amino groups are particularly well known. For example, *N,N*-dimethylamino propylacrylamide(DMAPAAQ) is a well-known quaternary ammonium salt-type cationic monomer. The quaternary amino group of DMAPAAQ is positively charged, which could attract counter anion Cl^−^ to maintain the charge balance [21]. Therefore, it is possible to either load the necessary anions or remove the harmful anions by exchanging the Cl^−^ ions with them.

**Figure 1:**
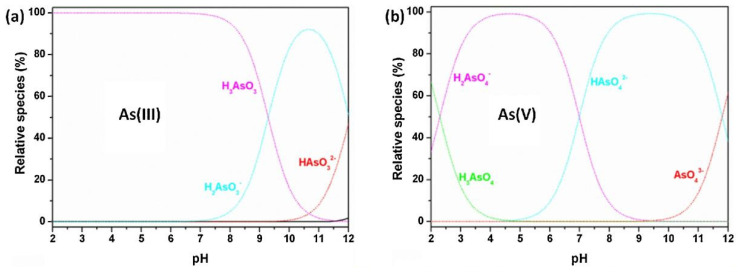
(**a**) As(III) and (**b**) As(V) speciation as a function of the solution pH [24].

The main forms of As in the groundwater are arsenic acid (H_3_AsO_4_) and arsenous acid (H_3_AsO_3_), which exist as molecules and exhibit negatively charged ionic states [22]. The As in groundwater often exists as arsenous acid as groundwater is in a reducing environment. It is known that As(III) has a lower adsorption capacity than As(V). For example, activated alumina (Al_2_O_3_), Fe compounds, such as Fe hydroxide, and Ce oxide [23] are known as As removers. However, the As ions are hardly adsorbed on the activated Al_2_O_3_. In addition, it is generally considered that the trivalent As (H_3_AsO_3_) is more toxic than the pentavalent As (H_3_AsO_4_) form. As illustrated in Figure 1, the anions of H_3_AsO_4_ exist over a wide pH range, whereas H_3_AsO_3_ does not dissociate in the acidic range and its anions exist only above pH 7, owing to which, it is less adsorbed. Therefore, it is necessary to oxidise the arsenite into arsenate in order to increase the removal efficiency. In other words, to increase the removal efficiency of the trivalent As, both an increase in the number of adsorption sites and the oxidation of As is essential.

Although the As adsorption method is used to remove As (III), most adsorbents have a very low As removal efficiency because they have few adsorption sites and a small absorption capacity. This restricts its application in the water treatment. To resolve these problems, we propose a polymer gel adsorbent with a dense network structure and many adsorption sites. In the present work, a polymer hydrogel was prepared by radical polymerisation of DMAPAA-Q by carrying an oxidising agent (KMnO_4_, K_2_Cr_2_O_7_) to enhance the adsorption capacity of As by changing the valence. The synthesised D-Mn gel (DMAPAAQ gel carrying MnO_4_^−^) and D-Cr gel (DMAPAAQ gel carrying Cr_2_O_7_^2−^) were systematically characterised before and after the As adsorption. Their As (III) removal performances were evaluated via a batch adsorption experiment. The adsorption kinetics and isotherms were also investigated in detail.

## 2. Results and Discussion

### 2.1. Effect of pH of the As Solution on the Gel Adsorption

**Figure 2:**
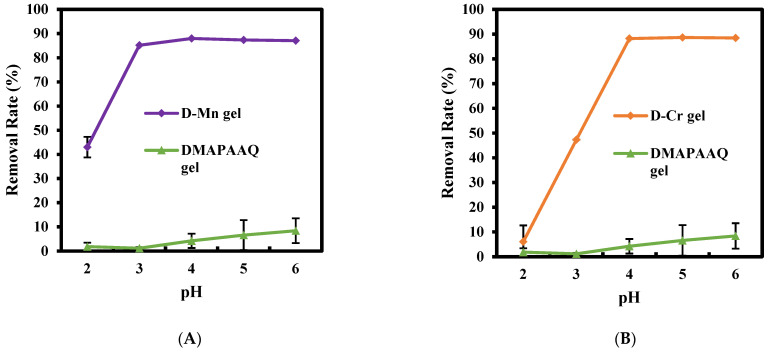
Effect of pH on the As(III) removal rate of the (**A**) D-Mn and (**B**) D-Cr gels.

For the adsorption of As(III) in aqueous environments, pH is a crucial parameter that affects the surface charge of the adsorbent as well as the conversion of the As species (Figure 1). Generally, different forms of As(III) are present in aqueous environments. H_3_AsO_3_ is dominant under the acidic conditions (pH < 7), while the anions, such as H_2_AsO_3_^−^, HAsO_3_^2−^, and AsO_3_^3−^ are dominant in alkaline environments [24]. To determine the effect of external pH on the As removal property of the gel, the As(III) adsorption by the D-Mn and D-Cr gels was first investigated in the pH range of 2−6 (Figure 2). As shown in Figure 2A,B, the DMAPAAQ gel removes a comparatively lesser amount of As (the removal rate of the DMAPAAQ < ~10%) than that of the D-Mn and D-Cr gels due to the formation of H_3_AsO_3_ under acidic conditions, which could interfere in the ion exchange between the Cl^−^ and As-based anions. Figure 2 shows that the As(III) removal rate of the D-Mn and D-Cr gels could still be maintained at a high level between pH 4 and 6. The adsorption process took place inside the gel, on which the effect of the external environment was minimal. Under acidic conditions, the redox reaction between MnO_4_^−^/Cr_2_O_7_^2−^ and As(III) in the gel is more likely to occur. Meanwhile a greater number of As(V) anions are produced by redox reactions, which eases As adsorption via the exchange of Cl^−^ and As(V) anions in the gels. Figure 2A also shows that the As removal rate of the D-Mn gel was significantly higher than that of the DMAPAAQ gel. This was because the Mn oxide supported in the gel oxidised As (III) into As (V), and also adsorbed the oxidation product, i.e., As (V).

**Scheme 1:**
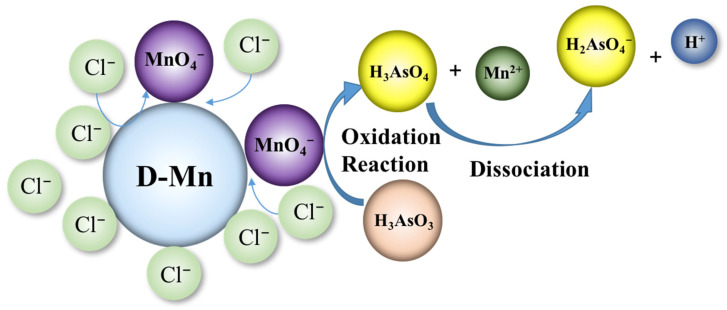
Schematic representation of the effect of high Cl^−^ ion concentration on the As adsorption of the D-Mn gel.

**Scheme 2:**
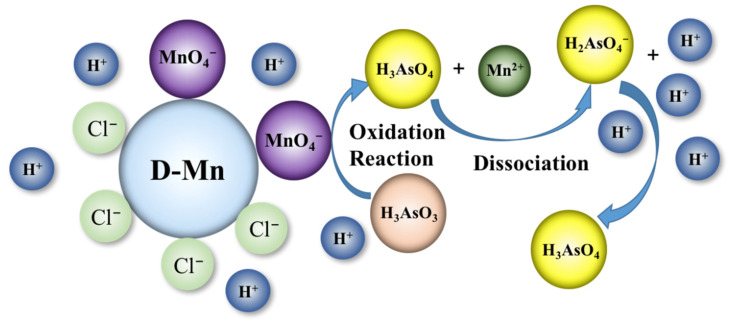
Schematic representation of the effect of high H^+^ ion concentration on the As adsorption of the D-Mn gel.

In addition, it was confirmed that the removal rate increased remarkably with the increasing pH, becoming nearly constant for pH > 3. However, when pH < 2, the As removal rate decreased significantly. This phenomenon could be attributed to two reasons: (1) large amounts of Cl^−^ ions derived from the HCl were used to adjust the pH, suppressing the formation of the oxidation product As(V) on the gel, as shown in Scheme 1; (2) when a large amount of H^+^ ions suppressed the dissociation of H_3_AsO_4_ and H_3_AsO_3_, the exchange of Cl^−^ and As (III) and As (V) was inhibited, thereby reducing the adsorption removal rate, as shown in Scheme 2.

### 2.2. Adsorption Isotherm

**Figure 3:**
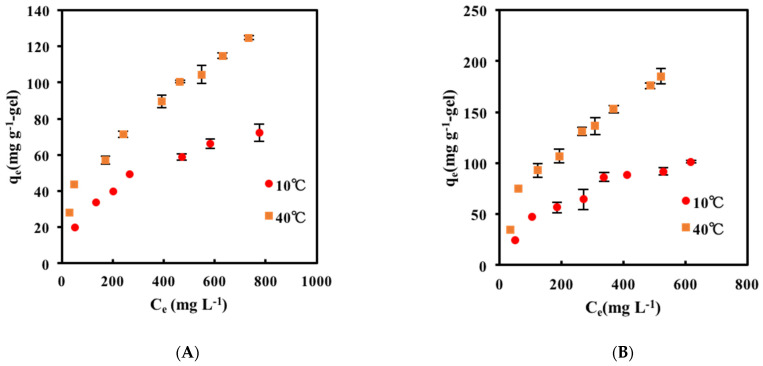
Isotherms of the As(III) adsorption in the (**A**) D-Mn and (**B**) D-Cr gels at different temperatures.

**Figure 4:**
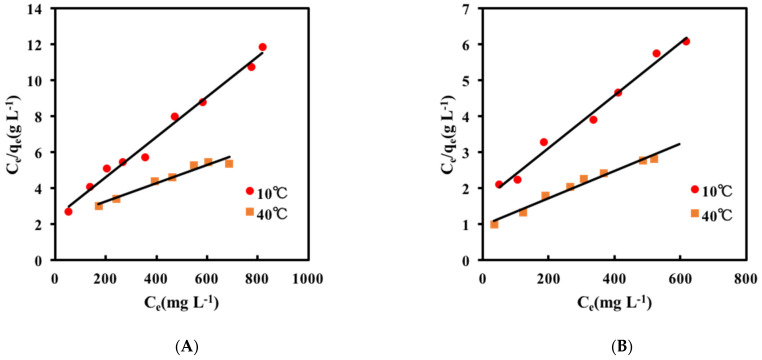
Langmuir isotherm plots for the As(III) adsorption in (**A**) D-Mn and (**B**) D-Cr gels at different temperatures.

Figure 3 shows the isotherms of As(III) adsorption by D-Mn and D-Cr gels at 10 °C and 40 °C, respectively, for 24 h (24 h is enough to ensure that the adsorption can reach an equilibrium state). The maximum amount of As adsorbed in the gel increased with the increasing temperature, as did the amount of As adsorbed in general. This enhancement in the adsorption process could be attributed to the progress of the redox reaction facilitated by the increase in temperature. The maximum adsorbed amount in the gel and the equilibrium adsorption constant could be calculated from the slope of the straight line and the intercept, respectively, according to the Langmuir isotherm adsorption Formula (2), as shown in Figure 4A,B.

Figure 4 shows the corresponding simulated results. The adsorption isothermal data were simulated using the Langmuir models. The parameters are listed in Table 1 and Table 2.

As mentioned above, the maximum amounts of As adsorbed by the two gels are 200 mg g^−1^-gel and 263 mg g^−1^-gel, respectively, which are considerably better than the maximum adsorbed amounts reported in previous studies, as listed in Table 3.

### 2.3. Adsorption Kinetics

The adsorption kinetics of D-Mn and D-Cr gels was investigated using both pseudo-first-order and pseudo-second-order kinetic models.

**Figure 5:**
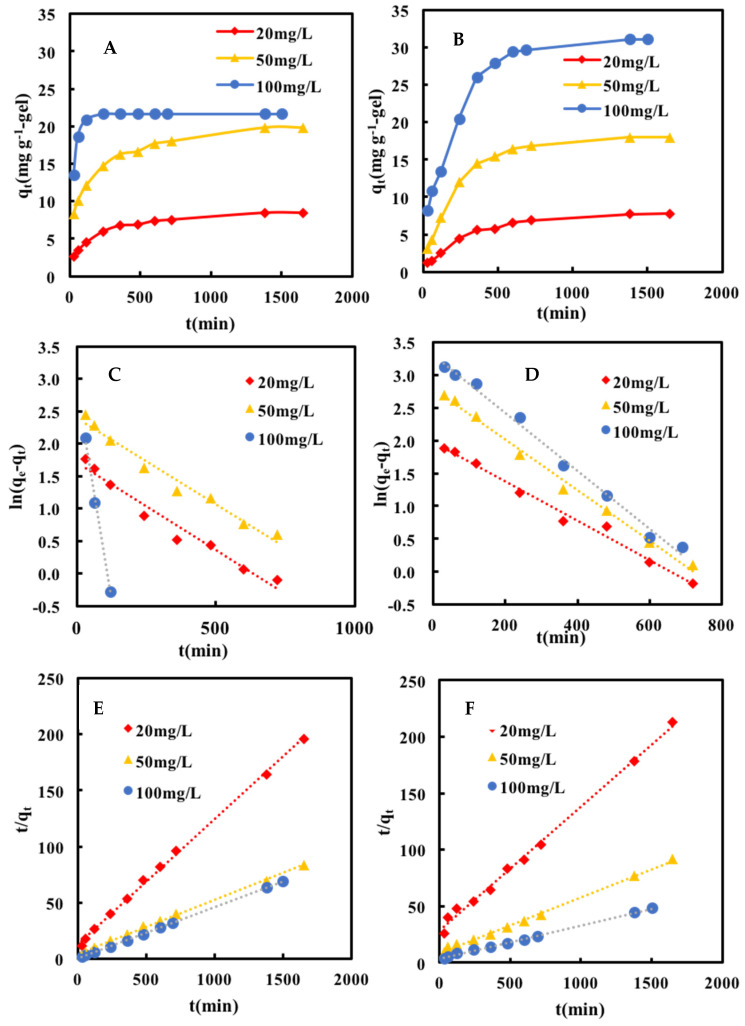
Adsorption kinetics of (**A**) D-Mn and (**B**) D-Cr gels; plots of the pseudo-first-order dynamic model of (**C**) D-Mn and (**D**) D-Cr gels; plots of the pseudo-second-order dynamic model of (**E**) D-Mn and (**F**) D-Cr gels.

The initial concentrations for the kinetic studies were set at 20 mg L^−1^, 50 mg L^−1^, and 100 mg L^−1^. The adsorbent dosage was set to 2 g L^−1^, and the contact time range was set at 30–1650 min. Figure 5A shows that the reaction reached equilibrium after 20 h for the initial concentrations of 20 mg L^−1^ and 50 mg L^−1^. However, at an initial concentration of 100 mg L^−1^, the adsorbed amount increased rapidly in the first hour, and the reaction reached equilibrium within the following 3 h. The figure suggests that the reaction rate increased significantly with the increasing initial concentration. Therefore, the MnO_4_^−^ ions were converted into MnO_2_ at low As(III) concentrations, and MnO_2_ could itself continuously adsorb As(III) after the redox reaction. Meanwhile, the MnO_4_^−^ ions underwent a vigorous redox reaction and were directly reduced to Mn^4+^ in a highly concentrated As solution. This is because the oxidised trivalent As became pentavalent and was rapidly adsorbed by the amino group of the gel at that time.

Unlike the D-Mn gel, there was no significant change in the adsorption rate of the D-Cr gel at the different initial As(III) concentrations. This suggests that an increase in the initial concentration of the As solution did not change the reaction rate of the D-Cr gel. To elucidate the mechanism of As adsorption by the D-Mn and D-Cr gels, the adsorption behaviour was analysed using the pseudo-first-order and pseudo-second-order dynamics equations, as shown in Figure 5C–F. Since both the D-Mn and D-Cr gels showed higher correlations with the pseudo-second-order dynamics model than with the pseudo-first-order model, As adsorption by the D-Mn and D-Cr gels was considered to be dominated by chemisorption. The parameters are listed in Table 4 and Table 5.

**Figure 6:**
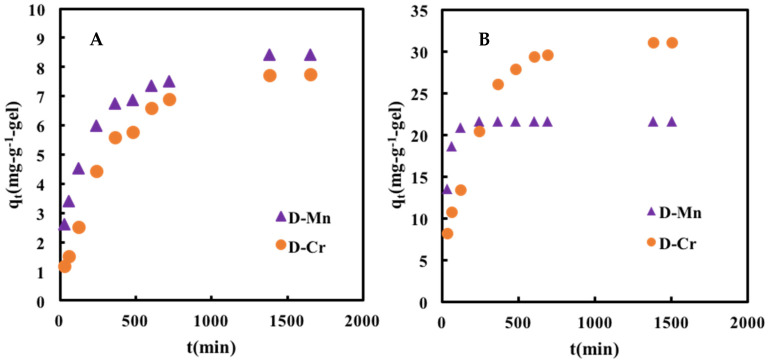
Comparison of the adsorption kinetics of the D-Mn and D-Cr gels at the initial As(III) concentration of (**A**) 20 mg L^−1^ and (**B**) 100 mg L^−1^, respectively.

Figure 6 shows the changes in the amount of As adsorbed on the gel with time at initial concentrations of 20 mg L^−1^ and 100 mg L^−1^. This figure shows that the adsorption performance of the D-Mn gel is superior to that of the D-Cr gel at the low As concentrations, which could be attributed to the ability of the MnO_4_^−^ ions to form MnO_2_ after the redox reaction, and continuously adsorb As even at low As concentrations. Meanwhile, the adsorption capacity of the D-Cr gel is much higher than that of the D-Mn gel, but the rate at which the latter reaches equilibrium is higher in the high-concentration As solutions. This is because the oxidising property of the MnO_4_^−^ ion is higher than that of the Cr_2_O_7_^2−^ ion in a neutral environment (standard oxidation reduction potential: KMnO_4_ (+1.51 V) > K_2_Cr_2_O_7_ (+1.35 V)).

### 2.4. Adsorption Thermodynamics

The mechanisms of the As(III) adsorption on the gels were investigated by calculating the thermodynamic parameters, such as adsorption enthalpy (Δ*H*), adsorption free energy (Δ*G*), and adsorption entropy (Δ*S*).

**Figure 7:**
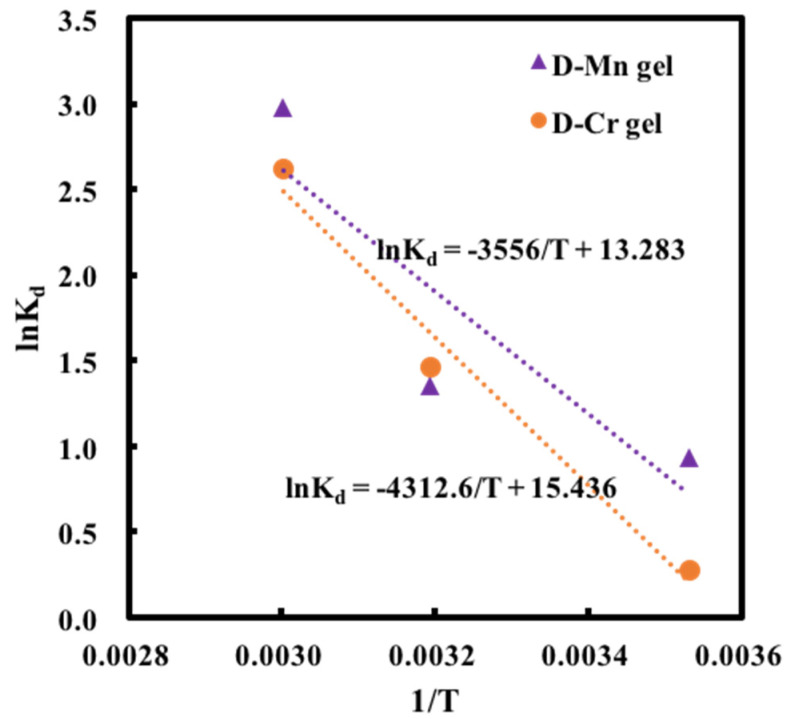
Effect of temperature on the distribution of the adsorption coefficient of the D-Mn (triangles) and D-Cr gels (circles).

Thermodynamic analyses of the adsorption experiments were conducted to elucidate the associated adsorption mechanisms. Experiments were conducted at the temperatures of 10, 40, and 60 °C to confirm the effect of temperature on the amount of As adsorbed in the gel (Figure 7). The figure was obtained by plotting the results calculated by the thermodynamic equation, and then, using other thermodynamic equations, the Δ*H* and Δ*S* values can be calculated. The results of the calculations are listed in Table 6 and Table 7, wherein Δ*G* < 0 suggests that the adsorption process could be performed naturally. The higher the reaction temperature, the greater is the decrease in Δ*G*. Therefore, as the temperature increased, the spontaneous reaction was facilitated to a certain extent. Since both Δ*H* and Δ*S* are > 0, the adsorption process could be interpreted as an endothermic chemisorption.

### 2.5. Effect of Co-Existing Ions on the As Removal Property of the D-Mn and D-Cr Gels

In wastewater treatment, various anions like HCO_3_^−^, SO_4_^2−^, PO_4_^3−^, and Cl^−^ are present in aqueous solutions. To determine the effect of the coexistence of other anions on the As adsorption, a coexisting ion adsorption experiment was conducted at an initial As(III) concentration of 20 mg L^−1^.

**Figure 8:**
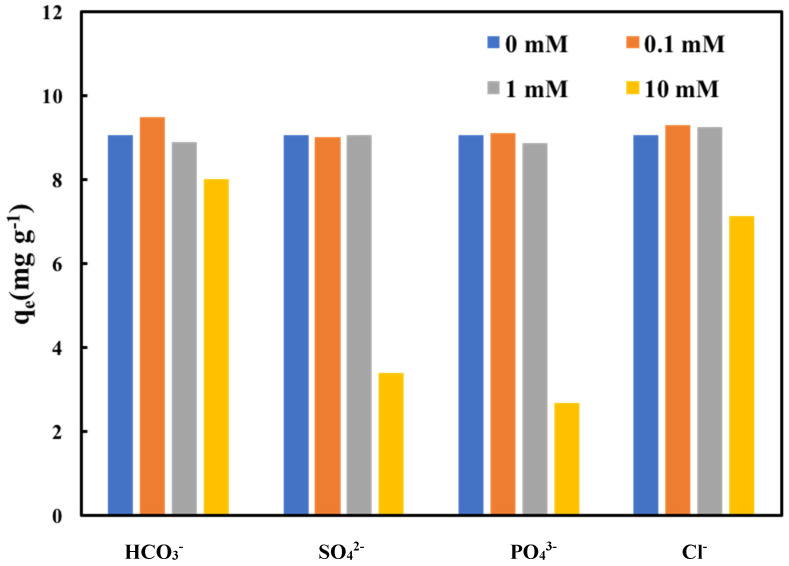
Effect of co-existing ions on the As removal property of the D-Mn gel.

**Figure 9:**
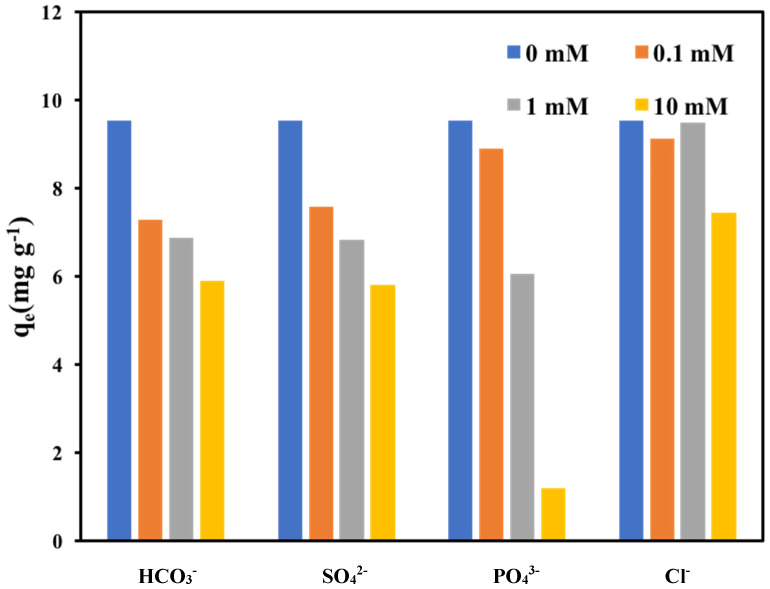
Effect of co-existing ions on As removal by the D-Cr gel.

Figure 8 shows that the amount of As adsorbed by the D-Mn gel varies considerably depending on the type of the co-existing ions. The influence on the As adsorption increased in the following order: PO_4_^3−^ > SO_4_^2−^ > HCO_3_^−^; therefore, the valence of the co-existing anions is considered to be strongly related to the amount of adsorbed As. Figure 9 shows that the adsorption capacity decreased with increasing valence of the co-existing anions. The gel also exhibited good stability with negligible change in adsorption at anion concentrations of 0, 0.1, and 1 mM. Therefore, this gel appears to be suitable for removing the trivalent As in the presence of the monovalent anions.

Similar to Figure 8, Figure 9 shows that the As adsorption capacity was significantly different in the co-existence of different ions. The valence of the co-existing anions appears to be closely related to the amount of As adsorbed: the adsorption capacity of the gel decreased with increasing valence of the co-existing anions. However, unlike that of the D-Mn gel, the adsorption capacity of the D-Cr gel gradually decreased with the increasing concentration of the co-existing ions, indicating that its stability is inferior to that of the D-Mn gel. However, in the presence of divalent anions, the D-Cr gel showed a larger adsorption capacity than the D-Mn gel. Therefore, the D-Cr gel is considered suitable for removing the trivalent As in the presence of the monovalent or divalent anions.

## 3. Conclusions

In summary, the polymer gel adsorbents (DMAPAAQ) carrying an oxidant (KMnO_4_/K_2_Cr_2_O_7_) were successfully synthesised (D-Mn and D-Cr gels). We demonstrated that a DMAPAAQ gel carrying an oxidant could significantly enhance the adsorption of As(III). Owing to the redox reaction with MnO_4_^−^ and Cr_2_O_7_^2−^ inside the gel, As(III) was oxidised to As(V), which increased the adsorption efficiency. As was also adsorbed onto MnO_2_ in the gel. The maximum adsorption capacities for As(III) of D-Mn and D-Cr gels reach 200 and 263 mg g^−1^-gel, respectively. The maximum adsorption capacities of the D-Mn and D-Cr gels were also larger than those of the other inorganic As adsorbents. The As adsorption in the D-Mn and D-Cr gels was dominated by chemisorption, as revealed by the adsorption kinetic analysis. The D-Mn gel appears to be suitable for removing As(III) in the presence of monovalent anions, while the D-Cr gel is considered suitable for removing As(III) in the presence of monovalent or divalent anions.

## 4. Materials and Methods

### 4.1. Reagents

*N,N*-dimethylamino propylacrylamide monomer (DMAPAAQ) was obtained by KJ Chemicals Co., Tokyo, Japan. Potassium dichromate (K_2_Cr_2_O_7_) and *N,N,N’,N’*- tetramethylethylenediamine (TEMED) were obtained from Nacalai Tesque, Inc., Kyoto, Japan, *N,N’*-methylenebisacrylamide (MBAA), ammoniumperoxodisulfate (APS), and potassium permanganate (KMnO_4_) were obtained from Sigma Aldrich Co., St. Louis, MO, USA. All the reagents were reagent grade and used as received. Aqueous solutions were prepared using distilled water (DW).

### 4.2. Synthesis of DMAPAAQ Hydrogel

In a 10 mL volumetric flask, 4.1342 g of DMAPAAQ (monomer), 0.1156 g of MBAA (cross-linking agent), and 0.0349 g of TEMED (accelerator) were dissolved in distilled water. APS (initiator, 0.0685 g) was dissolved in distilled water in a 5 mL volumetric flask (as shown in Table 8). N_2_ purge was performed for 30 min on each solution and the device to remove O_2_ to prevent the inhibition of radical polymerisation in the flask containing distilled water. After N_2_ purging, the initiator solution and monomer solution were mixed and stirred for 20 s. The obtained mixture was then injected into a gel-plate formation kit (AE-6401 1-mm Dual Mini Gel Cast, ATTO Corp., Tokyo, Japan). DMAPAA-Q and MBAA were polymerised at 25 °C for 24 h, following which, the gel was peeled off from the glass plate and cut into a 10 mm × 10 mm × 1 mm plate. The gel was washed with methanol for 24 h using a Soxhlet extractor (Asahi Glassplant Inc., Arao-city, Japan) to remove the unreacted monomers. After washing, the gel was dried at 25 °C for several days and then thoroughly dried in an oven at 50 °C.

### 4.3. Synthesis of the D-Mn Gel and D-Cr Gel

A 0.01 mol L^−1^ solution was prepared by dissolving 0.079 g KMnO_4_ in 50 mL distilled water. The DMAPAA-Q gel (0.2 g) was immersed in the prepared KMnO_4_ solution, and the mixture was kept at 25 °C for 24 h. The gel was then washed with ion-exchanged water for 24 h to remove the excess ions from the surface of the gel. The ion-exchanged water was then replaced several times in a fixed time interval (every 4~6 h). After washing, the gel was completely dried in a drying oven at 50 °C.

A 0.01 mol L^−1^ solution was prepared by dissolving 0.1471 g of K_2_Cr_2_O_7_ in 50 mL distilled water. In the prepared K_2_Cr_2_O_7_ solution, 0.2 g of DMAPAAQ gel was immersed, and the mixture was kept at 25 °C for 24 h. The gel was then washed with ion-exchanged water for 24 h to remove the excess ions on its surface. The ion-exchanged water was replaced several times at fixed time intervals in 24 h (every 4~6 h). After washing, the gel was completely dried in an oven at 50 °C.

### 4.4. Batch Adsorption Experiments

The batch adsorption experiments were performed in plastic vials (10 mL) with 2 g L^−1^ adsorbent dosage to investigate the effects of pH, reaction time, temperature, co-existing anions, and the effect of co-existing competing anions on As removal in the synthetic As(III) solution.

The effect of pH on the As(III) removal was explored at different initial pH levels (ranging from 2–6). The concentration of As(III) was 10 mg L^−1^, and the dosage of the D-Mn or D-Cr gels was 2 g L^−1^, respectively. The effect of the co-existing ions was investigated as follows: 20 mg of a given gel was added to 10 mL of As(III) solution (20 mg L^−1^) containing the ions HCO_3_^−^, SO_4_^2−^, and PO_4_^3−^, Cl^−^, wherein the initial concentrations of the co-existing ions were 0, 0.1, 1, and 10 mM. The mean values of all the data obtained were calculated from three individual measurements, and the standard deviations were added to some of the data.

The adsorption isotherms were measured to determine the As adsorption capacities of the adsorbents in the As(III) solutions with initial concentrations ranging from 10–1000 mg L^−1^ for 24 h. After reaching equilibrium, the D-Mn and D-Cr gels were separated to measure the residual concentration of As. The equilibrium adsorption capacity q_e_ was determined as follows:

(1) $$q_{e}=\frac{\left(C_{0}\;{-\;C}_{e}\right)V}{M}$$

where *C_0_* (mg L^−1^) and *C_e_* (mg L^−1^) are the initial and equilibrium As (III) concentrations, respectively, *V* (L) is the solution volume, and *M* (g) is the weight of the gel.

The Langmuir equations are expressed as follows:

(2) $$\frac{C_{e}}{q_{e}}=\frac{C_{e}}{Q_{m}}+\frac{1}{K_{L}Q_{m}}$$

where *q_e_* and *C_e_* are the adsorption quantity and equilibrium concentration of As(III) at the equilibrium state, respectively. *Q_m_* (mg g^−1^) is the maximum adsorbed amount, and *K_L_* (L mg^−1^) is the Langmuir adsorption isotherm constant.

The kinetics of As(III) adsorption was simulated using two mathematical models, namely the pseudo-first-order and pseudo-second-order kinetic models, which can be expressed as the following equations:

(3) $$ln\left(q_{e}{\;-\;q}_{t}\right){=lnq}_{e}{\;-\;k}_{1}t$$

(4) $$\frac{t}{q_{t}}=\frac{1}{k_{2}q_{e}{}^{2}}+\frac{t}{q_{e}}$$

Here, *q_e_* (mg g^−1^) is the equilibrium adsorption capacity, *q_t_* (mg g^−1^) is the adsorbed amount at time *t*, and *k*_1_ (min^−1^) and *k*_2_ (g mg^−1^ min^−1^) are the pseudo-first-order and pseudo-second-order rate constants, respectively.

Thermodynamic experiments were conducted by varying the initial concentration from 20 mg L^−1^ to 100 mg L^−1^ for 24 h at three different initial temperatures (10 °C, 25 °C, and 40 °C). The thermodynamic parameters for As(III) adsorption were quantified to explore the degree of spontaneity and heat exchange during the adsorption process. The standard Gibbs free energy Δ*G* (kJ mol^−1^), enthalpy change Δ*H* (kJ mol^−1^), and entropy change Δ*S* (J mol^−1^ K^−1^) were calculated as follows:

(5) $$K_{d}=\frac{q_{e}}{C_{e}}$$

(6) ΔG=ΔH − TΔS

(7) $$\Delta G=-RTlnK_{d}\;$$

(8) $${lnK}_{d}=\frac{\Delta S}{R}-\frac{\Delta H}{RT}$$

where *K_d_* is the distribution adsorption coefficient, *q_e_* (mg g^−1^) is the adsorption capacity per unit mass of the adsorbent, and *C_e_* (mg L^−1^) is the equilibrium As concentration.

All of the aqueous samples were filtered through a 0.22 μm membrane, and the concentration of the residual As was analysed by the inductively coupled plasma atomic emission spectroscopy (ICP-AES).

## Figures and Tables

**Table 1:** Langmuir model parameters for As(III) adsorption in D-Mn gel.

Temperature(°C)	Q_max_ (mg g^−1^-gel)	K_L_ (L mg^−1^)	R^2^
10	89	0.0005	0.9866
40	200	0.0002	0.9534

**Table 2:** Langmuir model parameters for the adsorption of As(III) in D-Cr gel.

Temperature(°C)	Q_max_ (mg g^−1^-gel)	K_L_ (L mg^−1^)	R^2^
10	137	0.0004	0.9855
40	263	0.0004	0.9758

**Table 3:** Comparison of the maximum As(III) removal capacity of related adsorbents.

Adsorbent	Initial As Concentration Range (mg L^−1^)	Adsorbent Dosage (g L^−1^)	Max. As(III) Adsorption Capacity (mg g^−1^)	References
D-Mn gel	1000−10	2	200	This work
D-Cr gel	1000−10	2	263	This work
Fe(III)/La(III)-chitosan	1−0.05	-	109	[19]
ZrPACM-43	100−10	13	41.48	[25]
Ceria-GO composite	200–0.01	0.5	185	[26]
Hydrous Cerium Oxide	100–1	0.5	170	[27]
CuO nanoparticles	400−200	0.08	39	[28]
TiO_2_ nanoparticles	90−5	1	31.35	[29]
Fe_3_O_4_-graphene composite	1–0.1	2	0.313	[11]

**Table 4:** Kinetic parameters of As(III) adsorption by the D-Mn gel.

Initial Concentration	Pseudo-First-Order Equation			Pseudo-Second-Order Equation		
	*k*_1_ (min^−1^)	*q_e_* (mg g^−1^)	R^2^	*k*_2_ (g mg^−1^ min^−1^)	*q_e_* (mg g^−1^)	*R* ^2^
20 mg L^−1^	0.0027	5.45	0.96586	0.00097	8.98	0.99830
50 mg L^−1^	0.0027	10.91	0.97547	0.00056	20.75	0.99822
100 mg L^−1^	−	−	−	0.00680	21.79	0.9999

**Table 5:** Kinetic parameters of As(III) adsorption by the D-Cr gel.

Initial Concentration	Pseudo-First-Order Equation			Pseudo-Second-Order Equation		
	*k*_1_ (min^−1^)	*q_e_* (mg g^−1^)	R^2^	*k*_2_ (g mg^−1^ min^−1^)	*q_e_* (mg g^−1^)	*R* ^2^
20 mg L^−1^	0.0030	7.23	0.98977	0.00043	9.08	0.99546
50 mg L^−1^	0.0039	16.27	0.99419	0.00029	20.20	0.99681
100 mg L^−1^	0.0044	27.46	0.99267	0.00023	34.01	0.99683

**Table 6:** Thermodynamic parameters of As adsorption in the D-Mn gel.

T(K)	$lnK_{d}$	**Δ*G* (kJ mol^−1^)**	**Δ*H* (kJ mol^−1^)**	**Δ*S* (kJ mol^−1^ K^−1^)**
283.15	0.93258781	−1.5815	29.565	0.110
313.15	1.35287955	−4.8815	29.565	0.110
333.15	2.97647589	−7.0815	29.565	0.110

**Table 7:** Thermodynamic parameters of As adsorption in the D-Cr gel.

T(K)	$lnK_{d}$	**Δ*G* (kJ mol^−1^)**	**Δ*H* (kJ mol^−1^)**	**Δ*S* (kJ mol^−1^ K^−1^)**
283.15	0.2777234	−0.3872	35.856	0.128
313.15	1.46240344	−4.2272	35.856	0.128
333.15	2.61940407	−6.7872	35.856	0.128

**Table 8:** Synthetic condition of DMAPAAQ hydrogel.

Component	Function	Molecular Weight (g mol^−1^)	Concentration (mol m^−3^)	Mass (g)
DMAPAAQ	Monomer	206.71	1000	4.1342
MBAA	Linker	154.17	50	0.1156
TEMED	Accelerator	116.21	20	0.0349
APS	Initiator	228.19	20	0.0685

## Data Availability

All data included in this study are available upon request by contact with the corresponding author.
